# Antibacterial, Antifungal and Antibiofilm Activities of Silver Nanoparticles Supported by Crude Bioactive Metabolites of Bionanofactories Isolated from Lake Mariout

**DOI:** 10.3390/molecules26103027

**Published:** 2021-05-19

**Authors:** Marwa Eltarahony, Amany Ibrahim, Hadeel El-shall, Eman Ibrahim, Fayez Althobaiti, Eman Fayad

**Affiliations:** 1 Environmental Biotechnology Department, Genetic Engineering and Biotechnology Research Institute (GEBRI), City of Scientific Research and Technological Applications (SRTA-City), New Borg El-Arab City 21934, Alexandria, Egypt; helshall@srtacity.sci.eg (H.E.-s.); esio2412@yahoo.com (E.I.); 2 Department of Biology, College of Science, Taif University, P.O. Box 11099, Taif 21944, Saudi Arabia; 3 Department of Biotechnology, College of Sciences, Taif University, P.O. Box 11099, Taif 21944, Saudi Arabia; faiz@tu.edu.sa (F.A.); e.esmail@tu.edu.sa (E.F.)

**Keywords:** antimicrobial, bioactive secondary metabolites, *Streptomyces* sp., nanobiotechnology, marine ecosystem, multidrug resistance

## Abstract

Lake Mariout is one of the polluted coastal marine ecosystems in Egypt which is considered to be a reservoir of serious effluents from different anthropogenic activities. Such selective pressure enforces indigenous microbial populations to acquire new advantageous themes. Thus, in this study, two *Streptomyces* strains were screened, from Lake Mariout’s sediment for bioreduction of 5 mM AgNO_3_. Both strains were identified molecularly; their biochemical and physiological characterization revealed their ability to secrete bioactive metabolites with antagonistic activity. The cultural and incubation conditions influencing AgNPs productivity were evaluated. Subsequently, the physicochemical properties of the biofabricated AgNPs were pursued. UV-Vis spectroscopy detected surface plasmon resonance at range 458–422 nm. XRD indicated crystalline, pure, face-centered cubic AgNPs; EDX demonstrated strong silver signal at 3.5 keV. Besides, FT-IR and TGA analysis unveiled self-stabilization and functionalization of AgNPs by bioorganic molecules. However, electron microscopy micrographs depicted numerous uniform spherical AgNPs (1.17–13.3 nm). Potent bactericidal and fungicide activity were recorded by zone of inhibition assay at 50 μg/mL. Further, the antibiofilm activity was exerted in a dose-dependent manner. Moreover, the conjugation of AgNPs with the crude bioactive metabolites of both bionanofactories ameliorated the antimicrobial potency, reflecting a synergistic efficiency versus examined pathogens (free-living and biofilm).

## 1. Introduction

Lake Mariout is one of the major lagoons in Egypt, its shore occupied by aquaculture and fishers. Topographically, it is located in Alexandria city, with coordinates that lie between latitude 31°9′11″ (N), longitude 29°53′55″ (E); it covers 50 km^2^ area with water depth range 0.6–1.5 m [1]. Based on artificial embankments, this shallow brackish lake is composed of four basins separated by rods, including main basin, south basin, east basin and the aquaculture basin. Through three main inflows (El-Qalaa, El-Umum and El-Nubariya) and other small outfalls from water treatment plants and the effluent drains from the petrochemical area, the lake receives substantial industrial, agricultural and municipal discharges that degrade the quality of the lake water [2,3]. Presently, several investigations have assessed its water quality by determining several parameters such as eutrophication status, heavy metals content, microbial diversity, salinity and inorganic matter contents as well [1,4,5]. However, the heavy metals concentration in Lake Mariout sediments seemed to be higher than those recorded in other Egyptian lakes and so it was referred to as the most anthropogenically polluted and eutrophic wetland in the Nile Delta [5].

Nonetheless, such intensive doses of heavy metals in conjugation with other anthropogenic organic and inorganic pollutants compel the indigenous living communities (microbial, planktonic, aquatic fauna) to acquire new additive functionalities for maintaining their stability either by resistance or resilience. Microbially, this adaptation phenomenon mediates via physiological modifications (induction/derepression of enzymes) and genetic mutation mechanisms that guarantee their coping with such a wide range of stressors [6,7]. Interestingly, several physiological adaptation strategies could be followed by microbes, including extracellular precipitation, intracellular bioaccumulation, cell surface biosorption, enzymatic oxidation/reduction and metal efflux [8]. It is worth mentioning that such bacterial means for metal detoxification participate mainly in the bottom-up approach of nanoparticles (NPs) preparation. The biological synthesis of metal NPs is deemed to be an essential building pillar of green nanotechnology applications. Bacteria-mediated synthesis of NPs, which is classified among such green synthesis approaches, poses the potential to substitute physicochemical means. It is characterized by an environmentally friendly nature, biocompatibility behavior, energy saving and lowering the risk of global warming [9,10,11].

By the virtue of small dimensions (≤100 nm) and high surface to volume ratio, magnetic and catalytic properties of metallic NPs and their oxides, including Ag, Au, Zn, Fe, etc., find their way into distinct bactericidal formulations for different applications such as food packaging, plant disease management, water disinfection and skin care products [12,13]. Recently, the combination of different antibiotics with biogenic metals NPs enhanced their antagonistic performance which is considered an upsurge opportunity to improve adjuvant or combination therapy against multidrug-resistant (MDR) pathogens [14,15,16].

Remarkably, actinomycetes are a prominent, phenotypically diverse clade among bacterial phyla which attract special biotechnological interest. Arguably, this filamentous bacterial group is an enormously important producer for powerful functional bioactive metabolites with a vast range of biological activities such as antimicrobial, antiviral, insecticides, herbicides, anticancer, immunomodulators and probiotic activity. Besides, it secretes a diverse array of enzymes which degrade and transform xenobiotic compounds and insoluble organic polymers into their simple substituents [17]. Moreover, it is resilient in extreme, hostile and contaminated ecosystems, representing an effective agent in the bioremediation process. Whereas, it produces spores, different types of metal chelators, metal uptake systems and extracellular polymeric substances (EPS) to adapt to such excessive pressure [17]. Recently, it was also listed as bionanofactories by endowing simultaneous reduction and functionalization for the as-synthesized NPs in one single pot and without additional successive steps in a cost-effective way. Whereas, it reduces metal salt and converts it to its nanostructure in oxidation–reduction reaction via their biomolecules, meanwhile encompassing the as-synthesized NPs and serving as capping and functionalizing agents [9,10,18,19].

Apparently, the polluted marine habitat could be considered to be a rich source for screening and isolation of unique actinomycetes strains with multicharacteristic features such as exhibiting new efficient bioactive metabolites, withstanding exceedingly high concentrations of pollutants and biosynthesis of NPs. Accordingly, selection pressure in polluted Lake Mariout met this multitarget aim. In the light of the above, our investigation focused on screening, isolation and characterization of AgNPs-producing actinomycetes isolates. Thereafter, the culture conditions influencing AgNPs synthesis were optimized. Further, the study was extended to examine the antibacterial, antifungal and antibiofilm efficiency of AgNPs alone and in combination with crude secondary bioactive metabolites.

## 2. Results

### 2.1. Screening, Isolation and Identification of AgNPs-Synthesizing Actinomycetes

**Figure 1:**
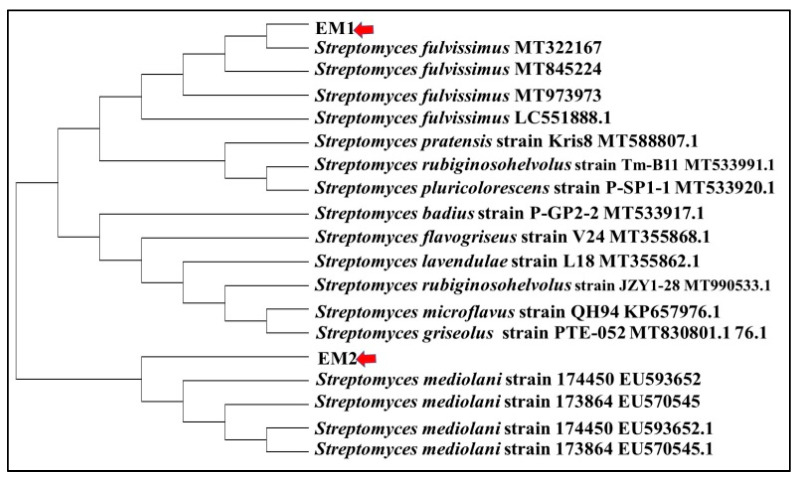
Phylogenetic tree of *S. fulvissimus* EM1 and *S. mediolani* EM2 based on 16S rRNA sequences analysis representing the relationship between two strains and other members of the *Streptomyces* spp.

The current investigation focused on the biosynthesis of AgNPs by actinomycetes isolates screened from one of the most polluted marine ecosystems in Alexandria. Only two isolates designated as EM1 and EM2 were obtained based on their capability to reduce 5 mM of AgNO_3_ to AgNPs. The isolates were subjected to taxonomic identification by the PCR amplification of 16S rDNA gene (approximately 720 bp), Blastn analysis, pairwise and multiple sequence alignment, which revealed ≥99% identity with the sequences of *Streptomyces fulvissimus* EM1 and *Streptomyces mediolani* EM2 and were deposited in NCBI GenBank under accession numbers KY964506 and KY964507, respectively. Phylogenetic relationship between selected strains and the closely related species was represented using the neighbor-joining (NJ) method as illustrated in Figure 1.

### 2.2. Characterization of AgNPs-Synthesizing Actinomycetes Strains

#### 2.2.1. Cultural and Morphological Characteristics

The development of pigments, morphology, forms of aerial hyphae and growth of vegetative hyphae of both selected strains were examined in different synthetic and complex media, which were tabulated in Table 1. Generally, both strains exhibited versatility in aerial, and substrate mycelia growth ranged from poor, moderate to good growth.

**Figure 2:**
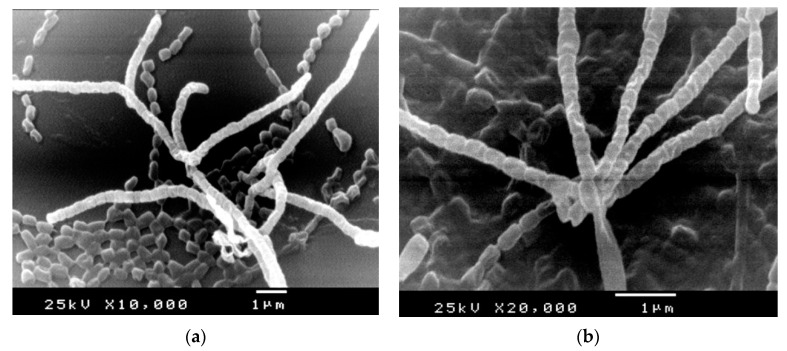
SEM micrographs showing morphology of aerial hyphae, fragmentation and rod-shape spores of *S. fulvissimus* EM1 (**a**) and *S. mediolani* EM2 (**b**).

The morphological properties of strains under study were visualized by SEM (Figure 2). As observed, well-developed aerial, vegetative hyphae were fragmented to smooth surface, long chain, small rods, non-flagellated spores. The micro morphological observations recorded the absence of both sclerotic granules and sporangia.

#### 2.2.2. Physiological Characteristics

There were common physiological features between *S. fulvissimus* EM1 and *S. mediolani* EM2, including growth in the pH range of 6.5–8.5 with optimum at pH 7. Good growth was exhibited in temperature range between 20–37 °C with the optimum temperature at 30 °C; both strains tolerated NaCl up to 5% with optimum growth at 2% salt, encompassing them in intermediate salt tolerance category. Besides, the growth was inhibited in the presence of phenol (0.1%) and was not influenced by sodium azide (0.01%). In addition, both strains assimilated different carbon sources such as D-glucose, starch, galactose, fructose, sucrose; both failed to assimilate citrate, mannose, xylose and cellulose. Regarding the biochemical characteristics, both strains displayed positive response to oxidase, catalase, urease and nitrate reductase. However, both recorded negative results for hydrogen sulfide production, gelatin liquefaction, DNase, cellulase and hydrolysis of protein and lipid. Generally, the loss and gain of biochemical capabilities depend on surrounding environment that influence the loss of some genes on account of the other to adapt the environmental stress [20,21].

#### 2.2.3. Characterization of Cell Wall Amino Acids

Chemotaxonomic analysis of *S. fulvissimus* EM1 showed the existence of LL- 2, 6 Diaminopimelic acid (LL-DAP) along with aspartic acid, leucine, valine, glycine, alanine, cystine, tyrosine, histidine, threonine and glycine, which ranged from trace to fair in the cell wall hydrolysate. Whereas, the cell-wall composition of *S. mediolani* EM2 contained LL- 2, 6 Diaminopimelic acid (LL-DAP) along with histidine and tyrosine. The findings of the work are consistent with [22,23].

#### 2.2.4. Screening of Bioactive Compounds

**Figure 3:**
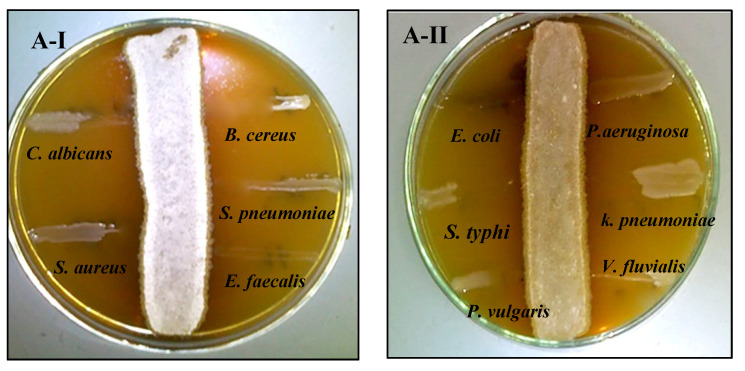

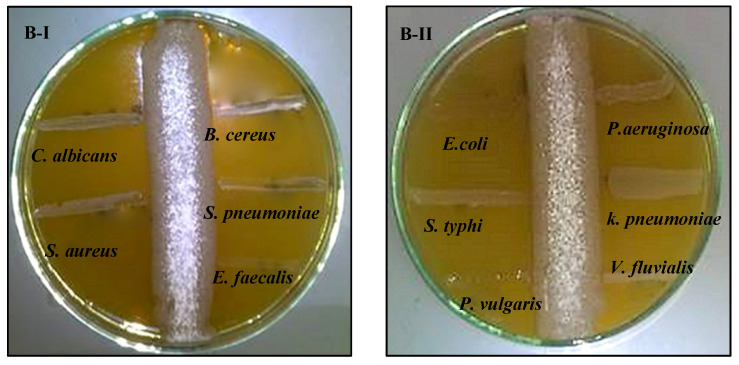
Antagonistic activity of *S. fulvissimus* EM1 (**AI–II**) and *S. mediolani* EM2 (**BI–II**) against pathogenic Gram-positive, Gram-negative bacteria and yeast.

The antagonistic activity of strains *S. fulvissimus* EM1 and *S. mediolani* EM2 was tested against various pathogens via streak plate approach. The antimicrobial activities of both strains were varied by suppressing the pathogenic microbes in different degrees. As displayed in Figure 3AI–II, there was no observed growth of the test pathogens, except *P. aeruginosa*, after 24 h near to the streaking of *S. fulvissimus* EM1 growth line, reflecting positive and high-score antimicrobial activity. That suggested a wide-spectrum nature for its bioactive compound. However, there was lower inhibitory effect of the bioactive metabolites produced by *S. mediolani* EM2, indicated by presence of growth of the test pathogens in the entire streak line, except *E. coli* (Figure 3BI–II).

### 2.3. Synthesis of AgNPs and Evaluation of Factors Enhancing Their Productivity

**Figure 4:**
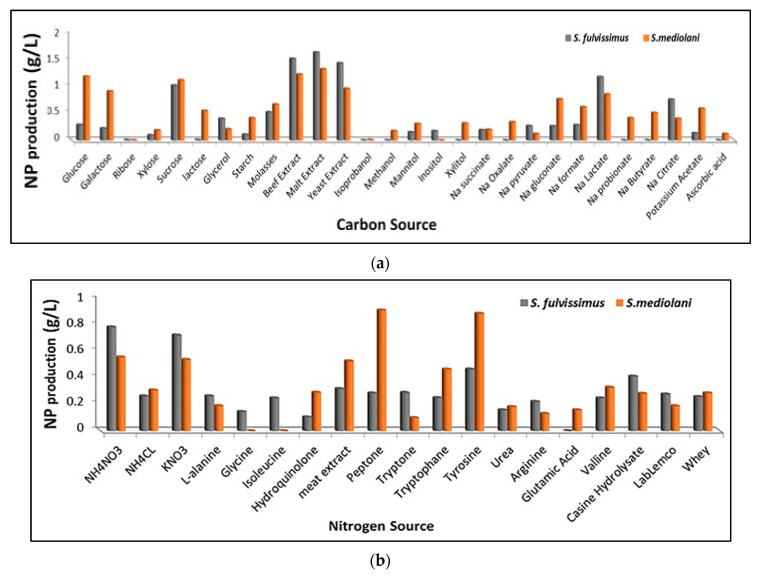

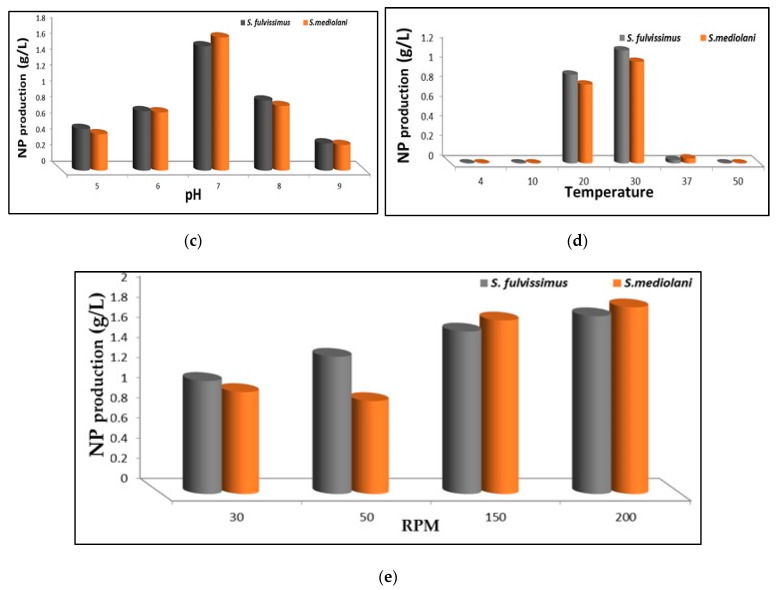
Effect of different carbon sources (**a**), nitrogen sources (**b**), pH (**c**), temperature (**d**) and RPM (**e**) on enhancing AgNPs productivity.

The preliminary assessment of silver precursor reduction and the consequent AgNPs production by both actinomycetes strains were identified by the change of color of the culture media and the change of actinomycetes pellets from yellow to black. Besides, no change in color was observed with negative control medium (without biomass) and biomass control medium (without AgNO_3_). The productivity of AgNPs was affected by several factors such as carbon sources, nitrogen sources, temperature, pH and RPM as illustrated in Figure 4a–e. These factors did not only support bacterial multiplication, but also enhanced the production and governed the rate of enzyme activity affecting the synthesis of silver nanoparticles [2,24].The data depicted graphically in Figure 4a revealed that the best carbon sources capable of promoting AgNPs biosynthesis by S. fulvissimus EM1 were complex organic groups including malt extract, beef extract and yeast extract; whereas, malt extract, glucose and sucrose were considered the optimum carbon sources for enhancing AgNPs productivity by *S. mediolani* EM2. Regarding the nitrogen sources (Figure 4b), ammonium nitrate, potassium nitrate, tyrosine and peptone improved biosynthesis of AgNPs by both strains. Other nitrogen sources such as amino acid, in particular, glutamic acid and glycine, showed negative impact on AgNPs production. Meanwhile, the optimum pH and temperature that uplifted AgNPs productivity were recorded at 7 and 30 °C for both strains (Figure 4c,d). Above and below these ranges, the productivity decreased. Moreover, the effect of the agitation speed in the production of AgNPs could be arranged in the following descending order; for both strains, 200 > 150 > 100 > 50 (Figure 4e), which was coincident with [25].

### 2.4. Activity Determination and Molecular Detection of Nitrate Reductase Enzyme

**Figure 5:**
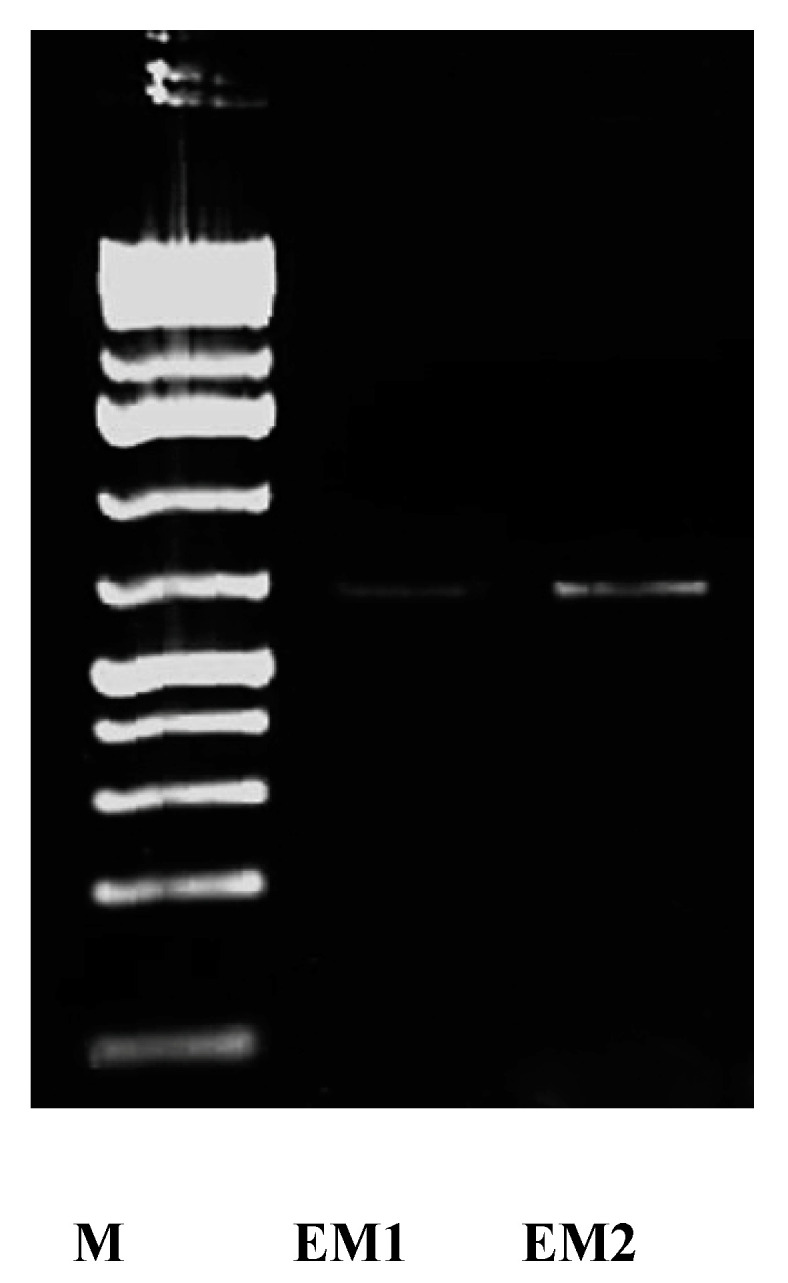
Agarose gel of the PCR products of NR gene of strain *S. fulvissimus* EM1 and *S. mediolani* EM2; Lane M: 1 Kb ladder mix (GeneRuler™ Fermentase).

In this study, the presence of NR enzyme was confirmed at biochemical and molecular levels. The activity of the enzyme was detected during all stages of the growth and synthesis process, where it was recorded as 53 and 71.2 µmole/min/mL after 12 h incubation for *S. fulvissimus* EM1 and *S. mediolani* EM2, respectively. It reached the maximum activity at 42 and 54 h by 212 and 371.2 µmole/min/mL for *S. fulvissimus* EM1 and *S. mediolani* EM2, respectively. At molecular level, NR enzyme was successfully detected on 1.5% agarose gel at 650 bp for both strains (Figure 5).

### 2.5. Production and Physicochemical Characterization of Biosynthesized AgNPs

Under selected optimized growth conditions, including carbon source (malt extract and beef extract), nitrogen source (ammonium nitrate and peptone) and neutral pH, the highest reduction of 5 mM of AgNO_3_ by *S. fulvissimus* EM1 and *S. mediolani* EM2, respectively, was achieved. Both cultures were incubated under 200 rpm and 30 °C. In this bottom-up approach, the silver salt was reduced into their respective NPs. The purified AgNPs were subjected to the following techniques to identify their physicochemical properties.

#### 2.5.1. UV-Vis Spectroscopy

**Figure 6:**
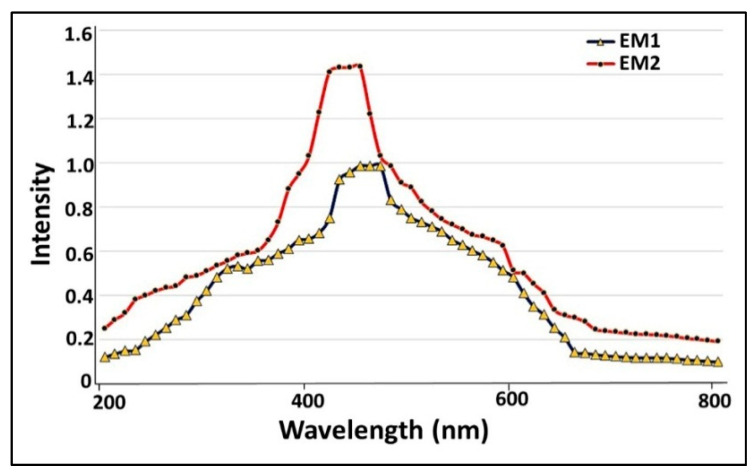
UV-Vis spectroscopy of microbially synthesized AgNPs by strains *S. fulvissimus* EM1 and *S. mediolani* EM2.

As a preliminary step to screen the optical and electronic features of examined nanoparticles, UV-Vis spectroscopy was employed. As demonstrated in Figure 6, a single surface plasmon resonance (SPR) band was localized at 458 and 422 nm for *S. fulvissimus* EM1 and *S. mediolani* EM2, respectively. Remarkably, the blackening of the cultural solution and pellets arose from excitation of longitudinal surface plasmon resonance (SPR), which is a unique feature for any material with a metallic nature. Nonetheless, it is affected by solution chemistry and synthesis method as reported by [26]. Likewise, [27,28] mentioned that the optimal peaks for green-synthesized AgNPs were located between 400–460 nm, which agreed with our results.

#### 2.5.2. Energy Dispersive X-ray Analysis (EDX)

**Figure 7:**
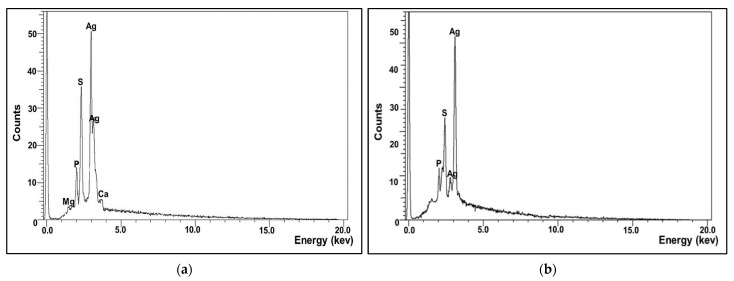
Elemental composition analysis of microbially synthesized AgNPs by strains *S. fulvissimus* EM1 (**a**) and *S. mediolani* EM2 (**b**).

The X-ray microanalysis gives qualitative as well as quantitative insinuation of elements that were involved in the fabrication of NPs. The elemental profile of the as-prepared AgNPs confirmed that silver is the main constituent element (Figure 7a,b). As shown, a strong unique elemental peak was noticed at 3.5 keV with weight percentages around 65.8%, which was ascribed to the SPR of the Ag nanocrystals. Besides, other elements such as P and S were detected in a considerable weight percentage which could be attributed to other microbial biomolecules tightly conjugated with AgNPs. The presence of such elements was commonly observed, particularly in green synthesized NPs, which suggested that they provide NPs with stabilization and functionalization [29,30].

#### 2.5.3. X-ray Diffraction (XRD)

**Figure 8:**
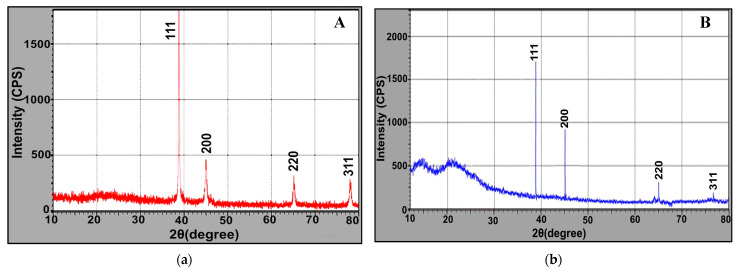
XRD (**a**,**b**) analysis of microbially synthesized AgNPs by strains *S. fulvissimus* EM1 and *S. mediolani* EM2, respectively.

The phase identity and crystalline structure of as-prepared AgNPs were verified by X-ray diffractogram. Figure 8a,b displayed four intense peaks at 2θ = 38.2°, 44.4°, 64.6° and 77.5° which correspond to hkl of (111, 200, 220 and 311) planes of face-centered cubic silver. These peaks matched with the standard pattern of JCPDS 0.4–0.783 [16]. Nonetheless, a little shift in the peak positions from XRD and small background at 2θ range 20–30° were shown, reflecting the presence of microbial proteinaceous residues associated with crystalline AgNPs. Evidently, our results are consistent with those obtained by [31,32].

#### 2.5.4. Fourier-Transform Infrared (FT-IR)

**Figure 9:**
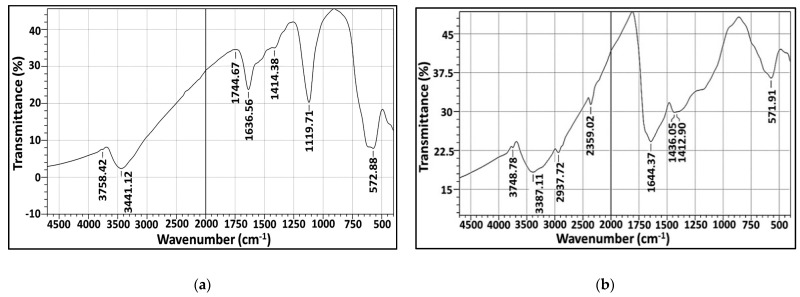
FT-IR (**a**,**b**) analysis of microbially synthesized AgNPs by strains *S. fulvissimus* EM1 and *S. mediolani* EM2, respectively.

This is a vibrational study applied to characterize the surface chemistry and identify the functional groups associated with AgNPs biosynthesized by both marine isolates under study. FT-IR spectrum of the freeze-dried powder of purified biogenic AgNPs is displayed in Figure 9a,b. As evident from the figure, there is the existence of common bands in the region of 3700 cm^−1^ which could be ascribed to stretching vibrations of O–H groups of water molecule as referenced by [33]. The vibration bands at 3444 and 3387 cm^−1^ could be attributed to primary amines (NH_2_) [34]; the carboxylic acid (OH) stretch was located at 2936 cm^−1^ [35]. However, peaks at 2356 cm^−1^ could be assigned to C-H asymmetric stretching vibration for aliphatic groups [36]. Meanwhile, the spectral peaks in around 1644, 1636 and 1744 cm^−1^ implied the presence of –C=C bond [36]. For the fingerprint region (600–1500 cm^−1^), a number of sharp bands were clearly observed. The absorbance bands centered at 1436, 1414 and 1412 cm^−1^ could be attributed to C-O stretch; the absorbance peaks at wavenumber 1119 cm^−1^ indicated the C–N stretching [37] vibration of primary aliphatic amines [38]. However, the incidence of band at 572 cm^−1^ revealed the (S–S) stretch band of protein and/or P–O–C groups in phospholipids [31,38]. Other studies reported that peaks at lower field in range 400–700 cm−1 reflected the metallic nature of any examined sample [39]. Generally, FT-IR study reflected the binding of protein, carbohydrates and phospholipids with AgNPs which contributed considerably to maintaining stability of AgNPs, by acting as capping and functionalizing agents and subsequently preventing them from agglomeration [40]. Apparently, the results of this study coincided with others [31,38,41].

#### 2.5.5. Thermogravimetric Analysis

**Figure 10:**
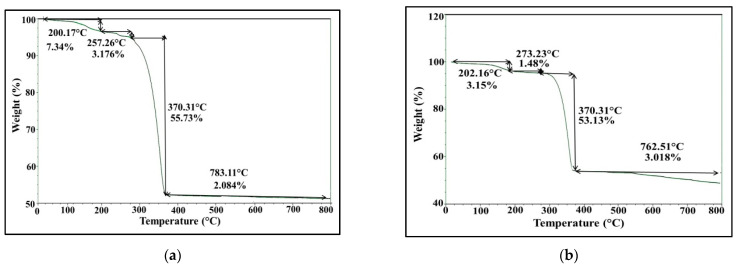
TGA (**a**,**b**) analysis of microbially synthesized AgNPs by strains *S. fulvissimus* EM1 and *S. mediolani* EM2, respectively.

TGA was conducted to study the behavior of biofabricated AgNPs at higher temperature through following up the change in their mass with temperature, which ultimately unveils their moisture content, thermal stability and the amount of organic layer surrounding them. The thermogravimetric curve represented in Figure 10a,b illustrates visible weight loss in four phases process during heating to 1000 °C in a controlled N_2_ atmosphere. First, the initial weight loss of AgNPs ranged from 3.5 to 4.5%, as was observed between 30 °C and 280 °C for *S. fulvissimus* EM1 and *S. mediolani* EM2, respectively. Such loss could be attributed to the evaporation of water molecules attached to AgNPs. In the second phase, 18.7 and 15.5% weight was lost between 280 °C and 620 °C for *S. fulvissimus* EM1 and *S. mediolani* EM2, respectively. The maximum weight loss by 31% was observed for both strains detected at the third phase between 620 and 770 °C, which was ascribed to the degradation of the organic residues such as proteins, polysaccharides and phospholipids conjugated to AgNPs. The final phase extended from 770 to 985 °C with weight loss assessed at 2.5%.

#### 2.5.6. Scanning and Transmission Electron Microscopy (SEM and TEM)

**Figure 11:**
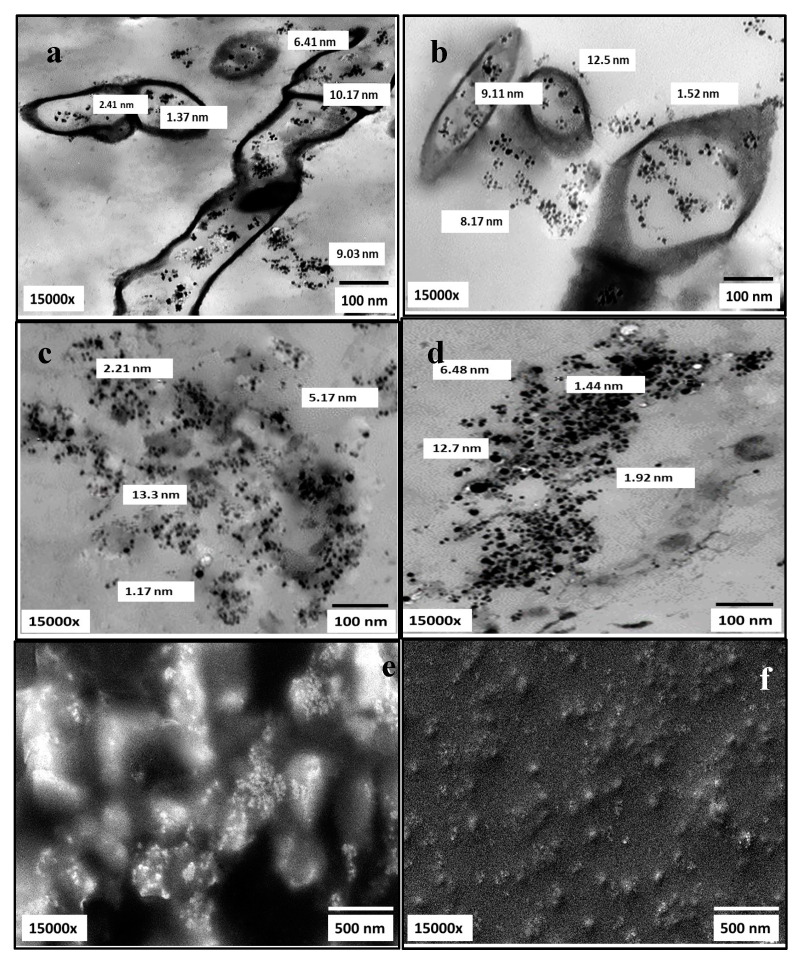
Electron microscopy micrographs (TEM; **a**–**d**) and (SEM; **e**,**f**) illustrating AgNPs producing nanofactories *S. fulvissimus* EM1 and *S. mediolani* EM2 (**a**,**b**) and AgNPs size and shape after extraction.

The morphology and size of AgNPs, besides their dispersion uniformity, were visualized by SEM and TEM micrographs. As elucidated in Figure 11, AgNPs biosynthesized by both strains appeared as numerous opaque electrons of uniform spherical NPs with particle size ranging from 1.17 nm to 13.3 nm in slight aggregates. As observed, AgNPs synthesized intracellularly and extracellularly. After extraction, they appeared well dispersed in agreement with [31,42].

### 2.6. Application of Biosynthesized AgNPs

#### 2.6.1. Antimicrobial Activity of Biosynthesized AgNPs against Free-Living Pathogens

In this study, the inhibitory potential of the as-prepared AgNPs was examined against various microbial groups by well diffusion assay, as listed in Table 2. Notably, the highest sensitivity to AgNPs was recorded by *B. cereus* by 0.8 ± 0.1 cm; however, the lowest sensitivity and the highest resistance were represented by *P. vulgaris* by 0.2 ± 0.0 cm. Hence, the image data revealed the sensitivity order of examined free-living pathogens against biosynthesized AgNPs as summarized as *B. cereus* ˃ *E. faecalis* ˃ *S. aureus* ˃ *E. coli* ˃ *S. typhi* = *P. aeruginosa* = *K. pneumoniae* ˃ *P. vulgaris*. Generally, the as-prepared AgNPs exerted higher suppression potential in the Gram-positive bacterial group relative to the Gram-negative group. More so, the fungicide potency of the biogenic AgNPs was displayed versus *C. albicans*, *A. bracelleuse* and *Alternaria* sp. Obvious zones of mycostasis were noted by 1.2 ± 0.05, 0.8 ± 0.05 and 0.8 ± 0.0 cm, respectively, Table 2.

#### 2.6.2. In Vitro Antibiofilm Efficiency of Biosynthesized AgNPs

Biofilm is a complicated structure of microbiome in which the microbial cells aggregate in mucilaginous-like matrix composed of polysaccharides, eDNA and proteins. It could be colonized in different biotic and abiotic surfaces, including natural, medical, industrial and food-processing devices which subsequently represent serious issues. By such a complex form of growth pattern, it could be recalcitrant to nutrient starvation, osmolarity, pH changes, mechanical forces and antibiotics [43]. As referenced by [44], it could tolerate up to 1000 times more than free-living pathogens by exerting multiple resistance mechanism. Hence, the employment of NPs as an antibiofilm is being unambiguously considered as an alternative solution. In this investigation, AgNPs were utilized in suppression of the biofilm synthesis by biofilm-producing prokaryotes (*P. vulgaris* and *B. cereus*) representing Gram-negative and Gram-positive classes, respectively. Moreover, *C. albicans* was examined as biofilm-forming eukaryotes. Obviously, remarkable progressive inhibition of biofilm formation was noticed with increasing of AgNPs concentrations (Table 3). Similar behavior of antibiofilm activity was observed in free-living, where Gram-positive biofilm exhibited higher susceptibility than Gram-negative biofilm against different concentrations of tested AgNPs. In fact, about 44.7 ± 2.8% of *B. cereus* biofilm was suppressed at 50 μg/mL and reached 91.7 ± 3.5% at 150 μg/mL; whereas, 21.5 ± 1.8 and 68.4 ± 4.1% were inhibited by the biofilm of *P. vulgaris* at exact concentrations, respectively. However, about 85.4% survival percentage was recorded by the biofilm of *C. albicans* at the lowest examined concentration.

#### 2.6.3. Synergistic Antimicrobial-Antibiofilm Activities of AgNPs Combined with Crude Metabolite of Selected Strains

Herein, the extracellular bioactive metabolites of both actinomycetes strains *S. fulvissimus* EM1 and *S. mediolani* EM2 were screened previously in Section 2.2.4. The combination between such crude bioactive metabolites and the biosynthesized AgNPs (50 and 100 μg/mL) was examined toward planktonic (Table 1) and biofilm (Table 2) lifestyles, respectively. As illustrated, with the selected AgNPs concentration, such combination strongly boosted antibacterial, anticandidal, antifungal and antibiofilm activity. It enhanced antagonistic activity among free-living pathogens by the range of 1.125-fold to 2.5-fold. Moreover, it significantly improved antibiofilm activity from 39.3 ± 3.3% to 71.8 ± 5.1 and 66.9 ± 3.4% toward *P. vulgaris* for combination with *S. fulvissimus* EM1 and *S. mediolani* EM2 crude metabolites, respectively. Whereas, the inhibition in biofilm formation by *B. cereus* increased significantly from 65.5 ± 2.1% to 93.4 ± 3.7 and 88.1 ± 3.9% for combination with *S. fulvissimus* EM1 and *S. mediolani* EM2 crude metabolites, correspondingly. Regarding *C. albicans*, the inhibition percentage increased by more than two-fold via such combination. Notably, the combination with crude metabolite excreted by strain *S. fulvissimus* EM1 displayed higher biocide potency than that shown by *S. mediolani* EM2.

## 3. Discussion

The emergence of multidrug-resistant pathogens (MDR) is an even more serious threat that is directly related to upsurges in mortality rate among severe nosocomial infections. Such a phenomenon appeared today as a result of globalization, an increase in growing population and immoderate, uncontrolled and multiple use of antibiotics and chemotherapeutics which ultimately restricted antibiotic therapy. Subsequently, success in finding new proceedings and new agents is the decisive solution for this issue. Therefore, exploring advantageous microbial groups with improved physiological capabilities from unique environmental habitats is urgently required. Additionally, the hybridization between nanotechnology and antimicrobial therapy is a promising approach to defeat this threat. Remarkably, the actinomycetes are classified among the most essential producers of efficient bioactive metabolites. As reported by [39], they produced more than 45% of known pharmaceutical products; additionally, they were categorized among nanoparticle producers, thus occupying a prominent site in both medical and biotechnological sectors.

Hence, the results of the present study were deemed characteristic where two marine strains were isolated from high salinity and contaminated lake and have the ability to produce active metabolites along with AgNPs. Thereafter, different nutritional parameters were evaluated to enhance the productivity of AgNPs. As carbon sources, malt extract and beef extract were selected; whereas, ammonium nitrate and peptone were utilized as nitrogen sources, in neutral pH and at 30 °C, to accelerate the reduction of 5 mM of AgNO_3_ by *S. fulvissimus* EM1 and *S. mediolani* EM2, respectively. Remarkably, several studies recorded the urgent requirement of actinomycetes for highly nutritive substrates that support metabolic reduction of the metals to their nanoform counterparts [45]. Reference [2] found that peptone-containing medium was the most significant nutritive factor for AgNPs production from S. viridodiastaticus. Besides, in agreement with our results, [2,24], declared that the internal environment of living cells is believed to be nearly neutral, so the activity of bacteria decreased as the pH deviates from neutral conditions. Reference [46] reported that the maximum AgNPs production was achieved at 25 °C, while at high temperature (40 °C), the enzyme activity decreased and hence the synthesis of AgNPs slowed down. However, References [47,48], found that optimum temperature for enzyme activity that enhanced AgNPs production was in the range of 20–30 °C.

However, the nitrate reductase enzyme, key enzyme that regulated the AgNPs formation, was determined successfully at molecular and biochemical levels. Interestingly, NADH-dependent nitrate reductase (NR) was proposed by various investigations to be involved substantially in NPs synthesis (Au, Fe, Cu, etc.) as a catalyzing biomolecule [49]. In general, the enzyme transforms nitrate to nitrite and the electron shuttle is stimulated to reduce the dissociated metal ions to their nanoform counterparts, in a continuous cycle of oxidation reduction reactions [50]. It is noteworthy to mention that the degenerated primers designed by [51] succeeded in detecting narG gene in several bacterial genera, including *Thermus* spp., *Streptomyces*, *Corynebacterium*, *Mycobacterium*, *Bacillus* spp. *Staphylococcus* spp., *Brucella* spp. and *Ralstonia* spp., which inferred the versatility of nitrate reductase function. It could be expressed variously for certain different functions in different organisms at different growth circumstances. Under microaerophilic conditions, it mediated the oxidation of NADH or any carbon source to generate proton gradient that enabled the production of ATP in *Campylobacter jejuni* [52]. Moreover, it catalyzed nitrate respiration in denitrification process anaerobically in *Paracoccus denitrificans* PD1222 [53]. Whereas, it dissipated excess electrons generated from growth on electron-rich substrates, under aerobic conditions, to maintain redox homeostasis in *Rhodobacter sphaeroides* [54]. Further, it played a crucial role in scavenging low concentrations of nitrate in *Shewanella* spp. [55].

By different characterization approaches, including UV-Vis spectroscopy, XRD, EDX, FT-IR, TGA, SEM and TEM, the optical, structural, elemental and morphological properties of the biosynthesized AgNPs were described. The biosynthesized AgNPs exhibited uniform spherical shape with slight aggregation associated with bioorganic molecules which eventually led to delaying their volatilization, stabilization and functionalization as implied by EDX, FT-IR and TGA. Such results matched those reported by [14,56,57,58]. Upon recruitment as antimicrobial agent, AgNPs displayed considerable and significant clear zones of inhibition in all examined pathogens, which were deemed good and effective as long as the zone exceeded 1 mm as highlighted by [59]. Notably, their effectiveness was more evident among Gram-positive group than Gram-negative one. That could be assigned to the structural variations of the outer cell wall. Whereas, Gram-positive bacteria were recognized by a thick peptidoglycan layer (20–80 nm) in the cell wall which exerts higher permeability than that of Gram-negative, which represents 7–8 nm. That makes Gram-positive bacteria more susceptible to biocidal agents. On the other hand, Gram-negative bacteria are characterized by the presence of an additional external lipopolysaccharide layer which might accumulate AgNPs in aggregates, preventing their entrance to the interior of the cell. In addition, Gram-negative bacteria pose a powerful multiple efflux pump system that externally expels any detrimental agents [59,60]. Remarkably, the efficiency of NPs versus pathogens was influenced by the microbial physiology, its metabolism, NPs dose, degree of contact and their diffusion rate. Apparently, our results match those reported elsewhere [16,60,61]. Moreover, the antifungal potency was pronounced against examined *C. albicans*, *A. bracelleuse* and *Alternaria* sp. Such fungicide efficacy could be explained by high damage of glycoprotein–glucan–chitin cross linkage which is the main constituent of the fungi cell wall. Consequently, the penetrated AgNPs caused metabolic collapse of the cellular biochemistry [11,31].

Interestingly, the higher surface area (surface/volume ratio) accompanied by small particle size NPs (10–80 nm) is considered the decisive factor in the biocidal activity. Such features enable a faster dissolution rate and more tight binding with the microbial cell which eventually lead to efficient cytotoxicity [62]. The antimicrobial activity of NPs could be summarized in the following steps. Whereas, it tightly attaches to the microbial cells, causing pits and holes in the cell wall, weakening membrane integrity and creating osmotic imbalance. Once NPs get inside the cell, it dissolves quickly in cytoplasmic solution, generating Ag ions that uplift reactive oxygen species level (ROS) and increase massive oxidative stress. Additionally, AgNPs interact selectively with active sites of biomolecules, causing protein malfunctioning and DNA disorder [40,63].

On the other hand, AgNPs showed antibiofilm capability in a dose-dependent manner, as the inhibition percentage decreases with continuous elevation in the treatment dosage. Our results concurred with the findings of [64,65]. The antibiofilm potency of biofabricated AgNPs could be exerted in multisuccessive stages begun by inhibition of the planktonic forms (initial stage of biofilm), followed by inhibition of exopolysaccharide formation which adheres to and aggregates the sessile cells (second stage), then passing through to blocking the quorum sensing activity [63].

Notably, several investigations have reported the enhancement of NPs efficiency in the combination of standard antimicrobial agents, hence, overcoming troubleshooting of multidrug resistance [15,16]. Therefore, the combination between the biogenic AgNPs and the crude bioactive metabolites of S. fulvissimus EM1 and S. mediolani EM2 was assessed and showed enhanced biocidal efficacy. Broadly, the highest antagonistic activity of such a combination could be attributed to the synergetic or additive effect of both constituents that disturb the microbial cell, frustrating its capability to mutate its genome for tolerating such condensed antimicrobial dose. Moreover, such a combination between bioactive metabolites and AgNPs might be directed concurrently to multiple active sites in the microbial cell, generating multiple damages such as cell wall deterioration, DNA/RNA inhibition and protein denaturation. That conclusion paves the way for promising avenues for its utilization in adjuvant therapy to overcome multiple antibiotic-resistant phenomena. Likewise, References [14,15,16] discussed similar findings. All of them found that the enhanced antagonistic activity of AgNPs was supported with certain antibiotics. Nonetheless, the exact constituent that prohibits the microbial growth in the crude bioactive metabolites of S. fulvissimus EM1 and S. mediolani EM2 will be optimized, extracted, purified and identified in an ongoing study. Generally, the current study implied the promising capability of AgNPs conjugated with crude metabolites in the management of infectious diseases, drug development and prevention of bacterial colonization.

## 4. Materials and Methods

### 4.1. Collection of Samples

Samples of water and sediments were collected from different places at Lake Mariout (main basin). The water samples were homogenously mixed, transported in sterile bottles, the different sediment samples were thoroughly mixed and placed in sterile plastic bags, then the samples were placed in ice until they were transferred to the laboratory. The samples were then stored at 4 °C before the actinomycetes were isolated.

### 4.2. Screening and Isolation of AgNPs-Producing Actinomycetes

Actinomycetes that have the capability to synthesize AgNPs were screened from the collected samples by serial dilution method on starch–nitrate medium, with the following components (g/L): 20 starch, 0.5 K_2_HPO_4_, 1 KNO_3_, 0.5 MgSO_4_.7H_2_O, 0.01 FeSO_4_ and 15 agar (for solid medium), pH 7. The medium was supplemented by different concentrations of AgNO_3_ (1.5, 3 and 5 mM). The inoculated plates were incubated at 30 °C for 7 days [39]. The actinomycetes isolates that had the ability to synthesize AgNPs caused blackening in media as a result of AgNO_3_ reduction. The selected colonies with different morphological characteristics were isolated and purified by streaking on the modified International Streptomyces Project 2 agar medium (ISP2). These isolated strains were regularly subcultured and stored on agar slants at 4 °C and hyphal fragments were preserved in 20% glycerol (*v*/*v*) at −80 °C until further use.

### 4.3. Molecular Identification of Actinomycetes Isolates Synthesizing AgNPs

The actinomycetes pellets of two isolates were obtained by their cultivation in shake flask of starch nitrate broth at 150 rpm at 30 °C for 4 days. Cells were collected by centrifugation and washed successive times with sterile phosphate buffer (pH 7). The bacterial genomic DNA of the selected isolates was extracted according to protocol followed by [15]. PCR amplicon was conducted using genomic DNA (0.1 μg) as a template and 20 pmol/μL of commercially synthesized 16S universal primer pairs 27f (5′AGA GTT TGA TCC TGG AG3′) and 1492r (5′TAC GGC TAC CTT GTT ACG ACT3′) in a 25 μL reaction volume containing 1 unit of Taq (Thermo Scientific), 15 μL of buffer and 2 μL of 10 mM dntp mix (Thermo Scientific). The PCR conditions began at 94 °C for 5 min as initial denaturation step, followed by 35 cycles of 94 °C for 20 s, 55 °C for 1 min and 72 °C for 1 min; the final extension step was at 72 °C for 10 min. Volumes of 5 µl of the PCR products were examined by loading on 1% agarose gel. The amplified PCR products were purified with GenEluateTM PCR Clean-Up Kit (Sigma) and sequenced. The phylogenetic position was inquired by comparing the procured sequences with the database sequences of NCBI. The sequences were deposited in the GenBank to obtain corresponding accession numbers. Evolutionary tree was inferred using the neighbor-joining technique by MEGA 6 software package.

### 4.4. Characterization of the Actinomycetes Isolates Synthesizing AgNPs

#### 4.4.1. Cultural Characteristics on Different Media

The macro-morphological and cultural characteristics of the selected isolates were studied by inoculation in the sterile International Streptomyces Project (ISP media): ISP-1 (casein yeast extract agar), ISP-2 (yeast extract, malt extract agar), ISP-4 (inorganic salt starch agar), ISP-5 (glycerol asparagine agar), ISP-6 (peptone yeast extract iron agar), ISP-7 (tyrosine agar). Moreover, their cultural behavior on LB, NB, glycerol-asparagine, casein-nitrate, starch-nitrate, starch-casein agar, Kuster’s agar and Bennet’s agar were tested [66,67]. Media were sterilized and poured into sterile plats. After solidifying the media, the culture of the selected isolates was streaked onto the medium surface and incubated for 7 days at 30 °C. Morphological characteristics such as colony characteristics, aerial hyphae type, vegetative hyphae growth, spore formation and pigments excretion were observed.

#### 4.4.2. Morphological Characteristics

The micro-morphological features of selected actinomycetes isolates, including hyphae fragmentation, spore chain, spore ornamentation and presence of sporangia were investigated and visualized by scanning electron microscope. At culture age 14 day, the specimens were prepared, fixed in glutaraldehyde (3%, *v*/*v*), washed and post-fixed in 1.5% osmium tetroxide for 2 h. The samples were washed, dehydrated by ethanol (40–100%), coated with gold and examined at 15–20 kV by SEM-JEOL JEM-1230-Japan.

#### 4.4.3. Physiological Characteristics

The growth of selected isolates under different pH (3, 5, 7, 9, 10 and 11), temperatures (10, 20, 30, 40 and 50 °C), salinity (1, 2, 3, 5, 7, 10 and 20%) was examined. The growth pattern in the presence of 0.1% phenol and sodium azide was recorded. However, the biochemical activities exhibited by both strains were determined by assimilation of different carbon sources and some enzymes. The carbon utilization test was performed using ISP-9 supplemented with 1% of the filter-sterilized D-glucose, sucrose, fructose, D-maltose, D-galactose, citrate, mannose, xylose, cellulose and starch. The mineral salt agar supplemented with starch, Tween 20, gelatin, casein, DNA, nitrate and urea was employed to examine the presence of amylase, lipase, gelatinase, protease, DNase, nitrate reductase and urease, respectively; besides, Kligler’s Iron Agar (KIA) was used to determine hydrogen sulfide production. Furthermore, oxidase and catalase were investigated according to [67].

#### 4.4.4. Characterization of Cell Wall Amino Acids

The chemical composition of the isolates cell wall was determined. Diaminobimlic acid isomers have been determined as described by [68].

#### 4.4.5. Screening of Bioactive Compounds

The perpendicular streaking method was employed to detect the presence of bioactive metabolites against some pathogens (*Pseudomonas aeruginosa* (ATCC 15442), *Escherichia coli* (ATCC 25922), *Salmonella typhimurium* (ATCC 14028), *Klebsiella pneumonia* (ATTC 700603), *Proteus vulgaris* (ATCC-8427), *Vibrio fluvialis* (ATCC 33809), *Bacillus cereus* (ATCC 33019), *Staphylococcus aureus* (ATCC 29213), *Enterococcus faecalis* (ATCC 29212), *Streptococcus pneumoniae* (ATCC 6303) and *Candida albicans* (ATCC 10231)). Briefly, the pure actinomycetes isolates were inoculated in a single line streak on nutrient agar and incubated at 30 °C for 4 days for the development of diffusible antimicrobial secondary metabolite. Thereafter, a single streak was inserted perpendicular to the actinomycetes line by each test pathogen in a parallel position to each other. The plates were incubated for 24 h at 37 °C [69].

### 4.5. Synthesis of AgNPs and Evaluation of Factors Enhancing Their Productivity

The biosynthesis process was initiated by inoculating 7 discs (9 mm diameter) taken from 72 h cultures of selected isolates in starch nitrate broth supplemented with 5 mM of AgNO_3_. The inoculated cultures were incubated for 120 h at 30 °C in an orbital shaker at 150 rpm. For enhancing the productivity of AgNPs, different parameters were scrutinized, including temperatures (4, 10, 20, 30, 40 and 50 °C), pH (5, 6, 7, 8 and 9), agitation (30, 50, 150 and 200 rpm), carbon sources (monosaccharides: ribose, xylose, glucose, galactose; disaccharides: sucrose, lactose; polysaccharides: starch; complex organic: beef extract, malt extract, yeast extract; sugar alcohols: isopropanol, methanol, glycerol, xylitol, mannitol, inositol; organic salts: citrate, oxalate, lactate, formate, succinate, butyrate, pyruvate, propionate; agricultural wastes: molasses) and nitrogen sources (inorganic salts: NH_4_NO_3_, NH_4_Cl, KNO_3_, urea; amino acids: L-alanine, L-glutamic acid, glycine, iso-leucine, valline, arginine, l-tyrosine, tryptophan; complex organic compounds: peptone, Lab-Lemco, casein hydrolysate, meat extract; dairy product wastes: whey). The incubation was followed as described formerly. The AgNPs were harvested by centrifugation, washed and extracted from the cells through conjugation of chemical lysis and physical disruption by sonication for 10 min at 40–60% amplitude and frequency of 20 kHz with 0.6 s pulse rate. The lysed suspension was vortexed vigorously for 5 min to obtain a homogeneous mixture. The extracted NPs were weighted and subjected to physicochemical analysis after centrifugation, washing and drying.

### 4.6. Activity Determination and Molecular Detection of Nitrate Reductase Enzyme

Under optimized conditions, the nitrate reductase activity for both isolates was assessed, as it was considered to be the key enzyme governing the bioreduction process [70,71]. In brief, the pellets of bionanofactories were harvested, washed and disrupted using mild osmotic shock. The obtained slurry was centrifuged at 12,000 rpm, 10 °C for 20 min. The supernatant was utilized as crude enzyme in a reaction mixture containing 0.2 M phosphate buffer (pH 7.0), 20 mM KNO3, 5 mM Benzyl Viologen and 10 mM sodium dithionite. After incubation at 30 °C for 30 min, the reagent A (2% sulfanilic acid) and B (0.2% N,N-dimethyl-1-naphthylamine) were added to the reaction mixture for detecting nitrite liberated as a result of nitrate reduction. The pink color was measured spectrophotometrically at 540 nm. Nitrite standard curve was performed to determine the concentration of nitrite. Remarkably, one unit of nitrate reductase represented the amount of enzyme that catalyzes the production of 1 μmol of nitrite/min or 1 μmol of nitrate reduced/min under standard assay conditions [70].

Moreover, the nitrate reductase enzyme was detected at molecular level using specific primers narG-1960f (5′ TAYGTSGGSCARGARAA 3′) and narG-2650r (5′ TTYTCRTACCABGTBGC 3′) which correspond to the large subunit of nitrate reductase enzyme (narG) [27]. The reaction mixture of 50 μL contained 2 μL of 25 ng DNA, 5 μL of 10× PCR buffer, 2 μL of 10 mM deoxynucleotide triphosphate (dATP, dCTP, dGTPand dTTP) mix, 10 μL (20 pmol) of primers and 1 U of Taq-polymerase; the thermocycling conditions included 5 min at 95 °C, 35 cycles of 1 min at 94 °C, 30 s at 60 °C, 45 s at 72 °C, 10 min at 72 °C. The amplified products were visualized on 1.5% (*w*/*v*) agarose gels stained with ethidium bromide (0.125 μg/mL) along with 1 Kb ladder mix (GeneRuler™ Fermentase) as a DNA marker and photographed in the MultiImage light cabinet.

### 4.7. Production and Physicochemical Characterization of Biosynthesized AgNPs

The absorption properties of the as-synthesized AgNPs were assessed at room temperature by Labomed model UV-Vis spectrophotometer. The maximum surface plasmon resonance (SPR) was detected in a wavelength range of 200–800 nm, as a first step for monitoring AgNO_3_ reduction. The fingerprint identification of AgNPs was determined using X-ray diffractometer (Shimadzu 7000, USA) that operates with Cu Kα radiation tube (λ = 1.5406 Å). The voltage and electric current were 30 kV and 30 mA, respectively; the scanning rate 2°/min for 2θ angular range of 2° to 80°. The obtained XRD patterns were compared with JCPDS database to analyze the data. The elemental contents of examined AgNPs samples were conducted by EDX detector connected with SEM- JEOL JEM-1230, Japan. However, TEM and SEM-JEOL JEM-1230-Japan, operating at 200 kV, were used to describe the morphology and size of as-prepared AgNPs. In addition, the surface chemistry and functional molecules associated with the biosynthesized AgNPs were detected, after washing, drying, grinding with KBr and formulation into pellets, by Shimadzu FT*IR-8400 S, Japan. The FT-IR spectrum was scanned in the region of 4000 to 400 cm^−1^ wave number at a resolution of 4 cm^−1^. Moreover, the thermal behavior of biosynthesized AgNPs toward increasing in temperature was analyzed by TGA-50H, Shimadzu (Japan). The experiment was performed in a nitrogen atmosphere at temperature range of 35–1000 °C and with heating rate of 20 °C/min [64,65].

### 4.8. Application of Biosynthesized AgNPs

#### 4.8.1. Antimicrobial Activity of Biosynthesized AgNPs

The antimicrobial activity of the biosynthesized AgNPs was examined versus various microbial pathogenic groups by well-diffusion method (zone of inhibition, ZOI). The examined procaryotic groups included Gram-negative bacteria (*P. aeruginosa* (ATCC 15442), *E. coli* (ATCC 25922), *P. vulgaris* (ATCC-8427), *K. pneumoniae* (ATTC 700603) and *S. typhimurium* (ATCC 14028)), Gram-positive bacteria (*B. cereus* (ATCC 33019), *S. aureus* (ATCC 29213) and *E. faecalis* (ATCC 29212)); however, the eukaryotic pathogens encompassed yeast *C. albicans* (ATCC 10231) and molds (*Aspergillus brasiliensis* (ATTC -16404) and *Alternaria* sp.). About 1 × 10^8^ CFU/mL of freshly prepared pathogens was spread uniformly on the Müller-Hinton agar using sterile cotton swab. The sterile cork-borer (10 mm) was used to make even punctures which were loaded by 50 μg/mL of biologically synthesized AgNPs in distilled water. The bacterial plates were incubated at 37 °C for 24 h; whereas, fungal plates were incubated at 25 ± 2 °C for 4–5 days. Negative control wells were run in parallel, containing distilled water. Upon the end of incubation, the plates were investigated for the presence of inhibition zone surrounding each well, which were measured and expressed in centimeters (cm) [65].

#### 4.8.2. In Vitro Antibiofilm Efficiency of Biosynthesized AgNPs

The inhibitory activity of the biosynthesized AgNPs was evaluated using the microtiter plate assay (MTP). In 96-well flat bottom polystyrene titer plates, about 1 × 10^6^ CFU/mL fresh cultures of *P. vulgaris* (ATCC-8427), *B. cereus* (ATCC 33019) and *C. albicans* (ATCC 10231) were inoculated separately in each well containing 100 μL of Tryptone Soy Broth (TSB). About 50 μL of AgNPs solution (50, 100 and 150 μg/mL) was added to each well. Positive and negative controls were examined simultaneously, containing bacterial suspension and sterile media only, respectively; the plates were incubated for 24 h at 37 °C. After incubation, the plates were processed according to the procedure described by [65]. The absorbance of the attached cells was assessed at 595 nm by a microtiter ELISA reader (Tecan Infinite M200, Switzerland), and the biofilm inhibition percentage was calculated by the following formula:

Inhibition percentage of biofilm = [(A − A0/A) × 100](1)

where A represents the absorbance of the positive control and A0 the absorbance of the AgNPs-treated well.

#### 4.8.3. Synergistic Antimicrobial-Antibiofilm Activities of AgNPs Combined with Crude Metabolite of Selected Isolates

The combination of AgNPs and the crude bioactive metabolites excreted by selected isolates was evaluated against the previously mentioned pathogens, both prokaryotes and eukaryotes, either free-living or biofilm. The crude bioactive metabolites were prepared by inoculating actinomycetes isolates separately in 75 mL of ISP2 media containing the following ingredients (g/L); malt extract, 10.0; yeast extract, 4.0; dextrose, 4.0. The final pH was adjusted to be 7.0 ± 0.2. The flasks were incubated at 30 °C for 4 days in an orbital shaker at 150 rpm. At the end of the incubation period, the cultures were collected by centrifugation at 12,000 rpm for 20 min. The obtained cell-free supernatant, which represents the crude bioactive metabolite, was filter sterilized with a 0.22 μm syringe filter. The combination was employed by thorough suspending of 50 and 100 μg/mL of AgNPs in the sterile crude bioactive metabolites rather than distilled water for free-living and biofilm, correspondingly. All the procedures for well diffusion method and microtiter plate assay were accomplished as previously mentioned in details [15,16].

### 4.9. Data Analysis

All results displayed in this investigation were represented by the means of three independent replicates ± standard error of the mean (SEM). The antimicrobial and antibiofilm activity data were subjected to analysis of variance (ANOVA) by GraphPad Prism software. Tukey post hoc was employed to analyze the mean difference comparison between the treatments. In all analyzed data, a probability level of *p* ≤ 0.05 was considered for the significance of differences between values.

## 5. Conclusions

This study sheds light on the biosynthesis of AgNPs via two *Streptomycetes* sp. strains isolated from Lake Mariout in a simple, less expensive, efficient and eco-friendly approach. It is anticipated that isolation of actinomycetes from a polluted marine ecosystem will be considered to be useful in the discovery of characteristic strains that exhibit a dual and simultaneous role as secondary metabolites producer and bionanofactory. The impact of different nutritional parameters on productivity of AgNPs was examined. The synthesized AgNPs under optimum conditions were characterized using UV-Vis spectroscopy, XRD, EDX, FT-IR, TGA, SEM and TEM. By applying as antimicrobial agent, the biosynthesized AgNPs exhibited promising antagonistic activity versus wide spectrum of Gram-positive and Gram-negative bacteria. AgNPs’ recorded ZOI ranged from 0.2 ± 0.0 cm by *P. vulgaris* to 0.8 ± 0.1 cm by *B. cereus.* The sensitivity order of examined free-living pathogens against biosynthesized AgNPs is summarized as *B. cereus* ˃ *E. faecalis* ˃ *S. aureus* ˃ *E. coli* ˃ *S. typhi* = *P. aeruginosa* = *K. pneumoniae* ˃ *P. vulgaris*. Additionally, powerful antifungal and antibiofilm efficacy were observed. By combination of AgNPs with secondary bioactive metabolites of both strains, the antagonistic capability increased by 1.125-fold to 2.5-fold for free-living pathogens and more than two-fold for biofilm and fungal pathogens, reflecting potential application in overcoming microbial threats.

## Figures and Tables

**Table 1:** Cultural characterization of *S. fulvissimus* EM1 and *S. mediolani* EM2 on different media.

Strain	Medium Type	Growth	Aerial hyphae	Substrate hyphae	Pigments
*Streptomyces fulvissimus* EM1	LB	Good	White	Pale yellow	None
	Glycerol-Asparagine	Moderate	White	Pale yellow	None
	Casein-NO_3_	Good	White	White	None
	Starch-NO_3_	Good	Yellowish to white	Colorless to white	None
	Starch-casein agar	Poor to moderate	White	Colorless to white	None
	Kuster’s agar	Good	White	Yellow to brown	
	Bennet’s agar	Very good growth	White	Yellow to brown	None
	NB	Very good growth	White	Yellow	Pale gray
	ISP1	Good	Pale gray	Yellow-orange	None
	ISP2	Very good	Off-white	Colorless to white	None
	ISP4	Very good	Off-white	Pale gray	None
	ISP5	Very good	Off-white	Pale gray	None
	ISP6	Very good	White	Brown	None
	ISP7	Very good	Pink	Pale gray	-ve melanine
*Streptomyces mediolani* EM2	LB	No growth	None	None	None
	Glycerol-Asparagine	No growth	None	None	None
	Casein-NO_3_	Good	Pale yellow	White	None
	Starch-NO_3_	Good	Yellowish to white	Colorless to white	Yellow
	Starch-casein agar	No growth	None	None	None
	Kuster’s agar	No growth	None	None	None
	Bennet’s agar	Moderate	Transparent to white	White	None
	NB	Very good	White	White to yellow	None
	ISP1	Good	Pale yellow	Yellow	None
	ISP2	Very good	White	White to yellow	None
	ISP4	Good	Orange	Yellow	None
	ISP5	Good	Transparent to white	White	None
	ISP6	Good	Transparent to white	Pale gray	None
	ISP7	Very good	Pink	Pale gray	-ve melanine

**Table 2:** Antimicrobial activity of biologically synthesized AgNPs and synergistic effect in combination with crude bioactive metabolites of *S. fulvissimus* EM1 and *S. mediolani* EM2 against wide spectrum of free-living microbes.

Microbial Group	Pathogen	Inhibition Zone (cm)		
		AgNPs	Crude Metabolites (EM1) +AgNPs	Crude Metabolites (EM2) +AgNPs
**Gram-Negative**	*E. coli*	0.5 ± 0.05	1.1 ± 0.3	0.8 ± 0.1
	*S. typhi*	0.3 ± 0.0	0.5 ± 0.05 ***	0.3 ± 0.05
	*P. aeruginosa*	0.3 ± 0.0	0.4 ± 0.05	0.3 ± 0.05
	*P. vulgaris*	0.2 ± 0.0	0.6 ± 0.05 ***	0.4 ± 0.05
	*K. pneumoniae*	0.3 ± 0.0	0.4 ± 0.05	0.3 ± 0.02
**Gram-Positive**	*S. aureus*	0.5 ± 0.05	0.9 ± 0.1 *	0.7 ± 0.1 *
	*B. cereus*	0.8 ± 0.05 ***	1.5 ± 0.1 ***	1.2 ± 0.3 ***
	*E. faecalis*	0.7 ± 0.1	1.2 ± 0.2 *	1.0 ± 0.2 *
**Yeast**	*C. albicans*	1.2 ± 0.05	2.0 ± 0.3 **	1.4 ± 0.3 *
**Molds**	*A. brasiliensis*	0.8 ± 0.05 *	1.2 ± 0.2	1.0 ± 0.3
	*Alternaria* sp.	0.8 ± 0.0	1.2 ± 0.3	0.9 ± 0.2 *

All values were expressed as mean ± SEM. AgNPs were compared with all other treatments, with significance at *p*-value <0.05 *, <0.005 **, <0.0005 ***.

**Table 3:** Antibiofilm behavior of biologically synthesized AgNPs and synergistic effect in combination with crude bioactive metabolites of *S. fulvissimus* EM1 and *S. mediolani* EM2 against biofilm-forming microbes.

Biofilm Type	Inhibition %				
	AgNPs Concentration (μg/mL)			Bioactive Metabolite Combined with 100 μg/mL AgNPs	
	50	100	150	*S. fulvissimus* EM1	*S. mediolani* EM2
*P. vulgaris*	21.5 ± 0.65 *	39.3 ± 1.7 *	68.4 ± 0.8 *	71.8 ± 1.4 *	66.9 ± 1.4 *
*B. cereus*	44.7 ± 2.8	65.9 ± 4.4	91.7 ± 2.5 *	94.1 ± 3.5 *	87.6 ± 2.4 *
*C. albicans*	14.6 ± 2.1	26.4± 1.5	64.4 ± 2.3 *	64.3 ± 1.2 *	61.8 ± 1.3 *

All values were expressed as mean ± SEM. AgNPs were compared with all other treatments, at each concentration, with significance at *p*-value <0.05 *.

## Data Availability

The data presented in this study are available on request from the corresponding authors.

## Sample Availability

Samples of the compounds are not available from the authors.
